# Investigating the Correlation Between Gray Matter Volume Changes and Cognitive Function Among Alzheimer's Disease Patients: An MRI-Based Analysis

**DOI:** 10.62641/aep.v53i4.1914

**Published:** 2025-08-05

**Authors:** Quan Sun, Luping Ma, Lulei Zhao, Mengfei Ye, Shaofeng Zhu, Jie Zhou

**Affiliations:** ^1^Department of Radiology, Shaoxing Seventh People's Hospital, 312000 Shaoxing, Zhejiang, China; ^2^Department of Radiology, Affiliated Hospital of Shaoxing University, 312020 Shaoxing, Zhejiang, China; ^3^Department of Radiology, Ningbo Yinzhou No.2 Hospital, 315199 Ningbo, Zhejiang, China; ^4^Department of Psychiatry, Shaoxing Seventh People's Hospital, 312000 Shaoxing, Zhejiang, China

**Keywords:** Alzheimer's disease, nuclear magnetic resonance, gray matter, cognitive function, memory function

## Abstract

**Objective:**

Alzheimer's disease (AD) is a neurodegenerative disease characterized by progressive cognitive impairment and memory dysfunction. This study aims to explore changes of gray matter volume and their relationship with cognitive and memory function in AD patients using magnetic resonance imaging-based analysis.

**Methods:**

This retrospective study analyzed the clinical data from 80 AD patients (AD group) and 45 patients with mild cognitive impairment (MCI group) treated in the hospital between January 2021 and December 2022. Furthermore, 43 healthy adults (control group) were also included for comparison. All the participants underwent a brain magnetic resonance imaging (MRI) examination. These three groups were comparatively analyzed for brain MRI imaging characteristics, changes of gray matter volume, as well as their cognitive and memory functions. Based on gray matter volume, AD patients were divided into the low-volume (37 cases) and high-volume (43 cases) groups using the K-mean clustering method. Furthermore, changes of cognitive and memory function across these two subgroups were compared. The correlation among gray matter volume, cognitive, and memory function across AD patients was assessed using *Pearson* correlation analysis. Additionally, predictive abilities of gray matter volume in severe cognitive impairment were determined employing receiver operating characteristic (ROC) curve analysis.

**Results:**

The gray matter volume, percentage of gray matter volume, scores of mini-mental state examination (MMSE), and California verbal learning test (CVLT-II) were significantly decreased across the control, MCI, and AD groups (*p* < 0.05). Compared to the control group, gray matter volume was reduced in the AD and MCI groups. Compared to the high-volume group, gray matter volume, percentage of gray matter volume, scores of MMSE and CVLT-II were decreased in the low-volume group (*p* < 0.05). Gray matter volume and gray matter volume percentage were positively correlated with scores of MMSE, immediate memory, delayed recall, cue recall, and long-delayed recognition (gray matter volume *r* = 0.384/0.334/0.308/0.251/0.333; percentage of gray matter volume *r* = 0.584/0.319/0.299/0.257/0.298; *p* < 0.001). The area under curve (AUC) for gray matter volume and gray matter volume percentage in predicting severe cognitive impairment were 0.833 (95% CI: 0.747–0.919) and 0.810 (95% CI: 0.715–0.904), respectively (*p* < 0.001), with sensitivity of 95.24% and 90.48%, and specificity of 66.10% and 67.80%, respectively.

**Conclusion:**

MRI is a useful tool for evaluating changes of gray matter volume in AD patients. The changes in gray matter volume are strongly correlated with cognitive and memory functions, which serve as a reliable predictor of severe cognitive impairment in AD patients. Furthermore, MRI provides robust imaging evidence for identifying AD patients at risk of severe cognitive impairment.

## Introduction

Alzheimer’s disease (AD) is a neurodegenerative disease characterized by progressive cognitive dysfunction and behavioral impairment, primarily observed in the elderly [1]. The onset of AD is affected by genetic predisposition, lifestyle choices, and environmental factors, and the risk of developing AD gradually increases with individual age. AD causes cognitive and memory impairment in patients, significantly influencing daily functioning. Patients may experience behavioral and personality abnormalities, resulting in challenges in self-care. Furthermore, they are prone to complications such as malnutrition and pressure sores, posing significant burdens on their families [2, 3].

Currently, there is no specific drug that can cure or reverse AD symptoms. The available treatment approaches focus on symptoms management and alleviating AD progression through pharmacological, non-pharmacological, and psychological interventions [4]. Assessing the severity of AD is essential for guiding clinicians in developing personalized treatment plans. However, current assessment approaches rely on scale-based tools, which have limitations. The results of some scales are affected by patient’s educational background, resulting in low accuracy, while others are complicated and increase physician’s workload. Therefore, identifying more objective AD assessment indicators is crucial in improving diagnosis and treatment approaches.

Magnetic resonance imaging (MRI) is based on the interaction of a strong external magnetic field and the human body’s hydrogen nuclei, which respond to specific radio frequency pulses. This technique allows for obtaining detailed images, enabling doctors to identify and assess lesions, thereby helping in disease diagnosis and treatment [5]. Recent studies underscore the advantages of standardized MRI measurement in evaluating cognitive impairment in older individuals [6], as well as their application in detecting and assessing AD [7]. However, most studies have focused on the white matter and the hippocampus in AD patients [8, 9]. Gray matter is densely populated with neurons and plays a vital role in transmitting nerve impulses. Furthermore, it is increasingly recognized for its role in cognitive and memory functions [10].

Therefore, this study aims to explore the potential relationship between changes in gray matter volume and cognitive and memory functions in AD patients, hoping to provide imaging-based insights to support cognitive and memory function evaluation in AD.

## Methods

### Recruitment of the Study Participants 

This retrospective study analyzed the clinical data of 80 AD patients (AD group) and 45 patients with mild cognitive impairment (MCI) (MCI group) who were admitted to the Shaoxing No.7 People’s Hospital between January 2021 and December 2022. Furthermore, clinical data from 43 healthy adults (control group) during the same period were collected for comparison. All patients underwent brain MRI examination.

Inclusion criteria for AD patients were as follows: (1) Met the AD diagnostic criteria [11]; (2) reported memory loss; (3) aged ≥60 years; (4) with no significant heart or lung dysfunction; (5) voluntarily participated in the study. Exclusion criteria for AD patients: (1) with history of stroke; (2) with epilepsy, brain tumors, or other diseases affecting brain function; (3) with alcohol or drug dependence; (4) with inability to cooperate with the examination; and (5) with claustrophobia. MCI patients met the MCI diagnostic criteria [12] but did not fulfill the criteria for dementia. The control group included patients without memory impairment or cognitive dysfunction, regardless of gender, and shared the same exclusion criteria as the AD group.

This study was approved by the ethics committee of Shaoxing No.7 People’s Hospital (Approval number: No.2023-011-01) and was conducted following the principles of *the Declaration of Helsinki*. All participants were informed about the study and voluntarily signed the informed consent form.

### Brain MRI Examination 

All subjects underwent brain MRI examinations using the Achieva 1.5T MRI scanner (MINFOUND, Hangzhou, China). They received standard brain MRI sequence scanning with T1WI sequence set at TE 15 ms, TR 2600 ms, a layer thickness of 5.0 mm, an interlayer gap of 1.0 mm, 1 excitation, a FOV of 230 mm × 230 mm, and a matrix of 240 × 240, without any interval between scanning. All scans were performed by the same imaging physician.

The obtained MRI data were transferred to a workstation where gray matter volume was measured using voxel-based morphometry (VBM). The raw data of the structural image were converted into NIFTI format using SPM12 software (developed by Friston *et al*., University of London, London, UK). Furthermore, the DARTEL tool was used to align the image to the Montreal Neurological Institute (MNI) standard space, reducing individual differences. The preprocessed images were then segmented using the built-in segmentation tool and tissue probability template to isolate the gray matter, after which the segmented image was resampled to a voxel size of 1.5 mm × 1.5 mm × 1.5 mm).

Furthermore, the image was spatially smoothed using an 8 mm full-width at half maximum to increase the signal-to-noise ratio and image quality and, an isotropic Gaussian filter convolution was applied to obtain the gray matter volume. Considering the individual differences in gray matter volume, the percentage of gray matter volume relative to total brain volume was used for correction. All MRI data post-processing was performed in a double-blind manner by two experienced radiologists. Utilizing the gray matter volume as the classification target, the AD patients were divided into a low-volume group (37 cases) and a high-volume group (43 cases) using the K-means clustering method, with the optimal number of clusters determined using the elbow method.

### Cognitive Function Assessment

Cognitive function was assessed using the mini-mental state examination (MMSE) [13], which evaluates orientation, memory, attention, calculation, recall, and language abilities. The total score ranges from 0 to 30 points, with lower scores indicating cognitive dysfunction. A score <27 indicates cognitive impairment. Cognitive dysfunction was categorized as follows: mild (21 to 26 points), moderate (10 to 20 points), and severe (0 to 9 points), respectively.

### Memory Function Assessment

The memory function of the subjects was assessed using the California Verbal Learning Test-II (CVLT-II) [14]. The test contained five sets of words with different semantic categories, each containing three nouns. The subjects randomly read the words and were asked to recall them immediately after each test. The test was repeated three times, and the total number of words recalled across the three trials was documented as the immediate memory score. After an interval of 20 minutes, subjects were asked to recall the words again, and the number of words remembered was recorded as the delayed recall score. Furthermore, to evaluate cued recall, participants were provided with word clues, and the number of words recalled was documented. Additionally, fifteen words with similar semantics were mixed into the test mixed words, followed by a recognition task. The difference between correctly and incorrectly recognized subjects was recorded as the long-term delayed recognition score.

### Statistical Analysis 

Data was statistically analyzed using SPSS 22.0 software (IBM Corporation, Armonk, NY, USA). The Shapiro-Wilk method was used to assess the normality of measurement data. Normally distributed data with homogeneous variance were expressed as mean ± standard deviation ($\bar{\chi }$ ± s). One-way analysis of variance (ANOVA) was used for comparison among multiple groups, with Bonferrino correction method used for pairwise comparisons. The *t*-test was used for comparisons between the two groups. Categorical data were expressed as number of cases (%) and were analyzed using the χ^2^ test. The correlation between gray matter volume and cognitive and memory function in AD patients was analyzed using *Spearman *correlation analysis. The receiver operating characteristic (ROC) curve analysis was used to assess the predictive value of gray matter volume for severe cognitive impairment in AD patients. An area under the ROC curve (AUC) >0.7 indicated good predictive efficiency. The optimal ROC threshold and its corresponding sensitivity and specificity were determined by employing the Youden index. Statistical significance was set at α = 0.05.

## Results

### Comparison of Baseline Characteristics Among the AD, MCI, and Control Groups 

There were no significant statistical differences among the AD, MCI, and control groups regarding age, gender, education time, and the prevalence of hypertension, diabetes, smoking history, and drinking history (*p* > 0.05, Table 1).

**Table 1.** **Comparison of baseline characteristics among the AD, MCI, and control groups**.

Baseline characteristics		AD group (n = 80)	MCI group (n = 45)	Control group (n = 43)	*F*/χ^2^	*p*-value
Age (years)		66.14 ± 4.15	67.38 ± 4.33	66.82 ± 4.27	1.287	0.279
Gender					0.284	0.868
	Male	46 (57.50)	24 (53.33)	23 (53.49)		
	Female	34 (42.50)	21 (46.67)	20 (46.51)		
Education time (years)		12.51 ± 3.14	11.94 ± 2.87	12.03 ± 3.01	0.638	0.530
Hypertension		33 (41.25)	17 (37.78)	17 (39.53)	0.148	0.929
Diabetes		15 (18.75)	8 (17.78)	7 (16.28)	0.117	0.943
Smoking history		29 (36.25)	16 (35.56)	12 (27.91)	0.942	0.625
Drinking history		31 (38.75)	17 (37.78)	14 (32.56)	0.481	0.786

Note: AD, Alzheimer’s disease; MCI, mild cognitive impairment.

### Comparison of Gray Matter Volume Among the AD, MCI, and Control Groups

**Fig. 1.**
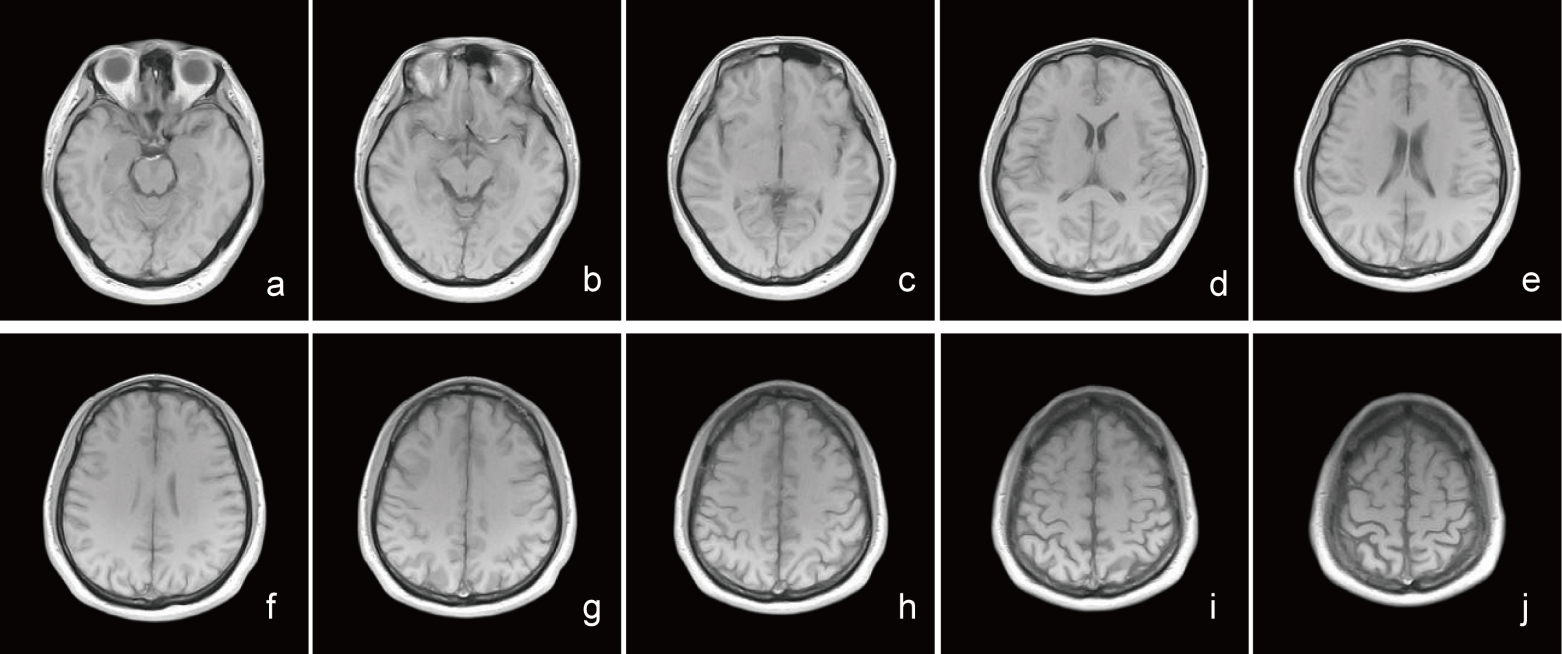
**The magnetic resonance imaging (MRI) T1WI sequence imaging of the brain of a healthy adult during physical examination**. (a–j) The T1WI sequence transverse imaging of a 78-year-old female with no memory loss. MRI shows no signs of reduction in brain gray matter volume.

**Fig. 2.**
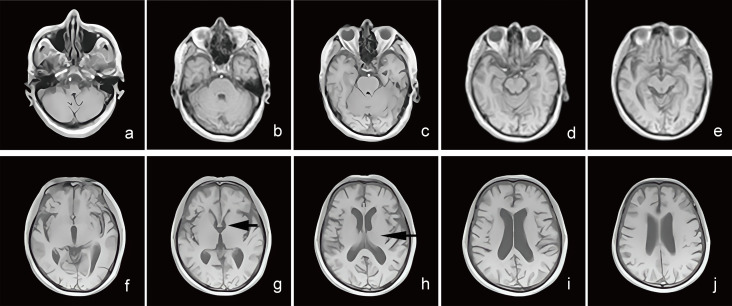
**Brain MRI T1WI sequence imaging of MCI patients**. (a–j) The T1WI sequence transverse imaging of a 79-year-old male, clinically diagnosed with MCI, with memory loss for more than half a year. (g,h) The black arrows indicate the reduction of gray matter volume in the temporal lobe and insular cortex.

**Fig. 3.**
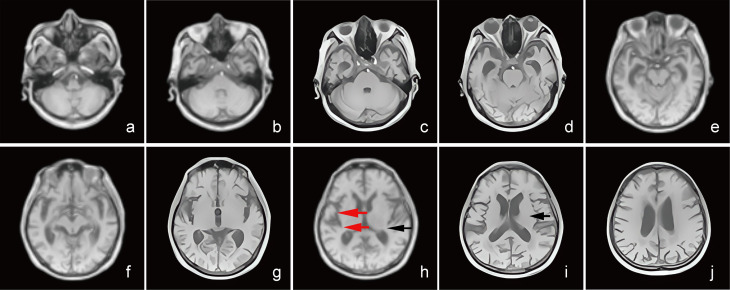
**Brain MRI T1WI sequence imaging of AD patients**. (a–j) The T1WI sequence transverse imaging of 78-year-old male, clinically diagnosed with AD, with memory loss for 1 year, forgetfulness, and inability to call the names of family members. (h,i) MRI shows significant reduction in the cortical gray matter as shown by the black arrows, and (h) the red indicates the reduction of the gray matter volume in the bilateral basal ganglia and thalamus.

The findings of the brain MRI T1WI sequence for the AD group, MCI group, and control group are shown in Figs. 1,2,3 and Table 2. The brain MRI T1WI sequence of healthy adults showed no signs of gray matter volume reduction. In the MCI groups, MRI scans showed localized gray matter atrophy, mainly distributed in the temporal lobe and insular cortex. The brain MRI T1WI sequence of AD patients revealed a reduction in cortical gray matter, along with gray matter volume decline in the bilateral basal ganglia and thalamus. Furthermore, compared to the control group, the AD and MCI groups demonstrated a significant reduction in gray matter volume and the percentage of brain gray matter volume (*p* < 0.05). Moreover, compared to the MCI group, the AD group exhibited further decline in gray matter volume and the percentage of brain gray matter (*p* < 0.05).

**Table 2.** **Comparison of brain gray matter volume and brain gray matter volume percentage among the AD, MCI, and control groups**.

Variables	AD group (*n* = 80)	MCI group (*n* = 45)	Control group (*n* = 43)	*F*	*p*-value
Gray matter volume (mm^3^)	703.16 ± 59.41*#	786.37 ± 62.73*	836.42 ± 68.42	68.795	<0.001
Gray matter percentage (%)	30.07 ± 3.02*#	33.86 ± 2.79*	37.16 ± 3.21	80.666	<0.001

Note: AD, Alzheimer’s disease; MCI, mild cognitive impairment; *p < 0.05 vs control group. #*p* < 0.05 vs MCI group.

### Comparison of MMSE and CVLT-II Scores Among the AD, MCI, and Control Groups

Compared with the control group, the MMSE scores and memory function-related scores were significantly reduced in the AD and MCI groups (*p* < 0.05). Compared to the MCI group, the AD group demonstrated a further decline in the MMSE scores and memory function-related scores (*p* < 0.05, Table 3).

**Table 3.** **Comparison of gray matter volume, MMSE score, and CVLT-II score among the AD, MCI, and control groups**.

Variables		AD group (n = 80)	MCI group (n = 45)	Control group (n = 43)	F	*p*-value
MMSE (points)		18.32 ± 3.31 *#	24.66 ± 2.43 *	28.94 ± 0.42	246.295	<0.001
CVLT-II scores						
	Immediate memory (minutes)	11.72 ± 2.96 *#	17.62 ± 2.84 *	24.83 ± 3.07	278.105	<0.001
	Delayed recall (points)	1.67 ± 0.37 *#	5.81 ± 1.11 *	11.72 ± 1.36	1640.465	<0.001
	Cued recall (points)	4.34 ± 0.83 *#	7.12 ± 1.83 *	12.08 ± 2.13	352.363	<0.001
	Long-delayed recognition (points)	4.98 ± 1.24 *#	8.33 ± 1.62 *	13.72 ± 1.37	558.901	<0.001

Note: AD, Alzheimer’s disease, MCI, mild cognitive impairment, MMSE, mini-mental state examination, CVLT-II, California verbal learning test, *p < 0.05 *vs* Control group, #*p* < 0.05 *vs* MCI group.

### Comparison of Brain Gray Matter Volume, MMSE Score, and CVLT-II Score Across AD Patient Subgroups

The brain gray matter volume, brain gray matter volume percentage, MMSE score, and CVLT-II score were significantly lower in the low-volume group than in the high-volume group (*p* < 0.05, Table 4).

**Table 4.** **Comparison of brain gray matter volume, brain gray matter volume percentage, MMSE score, and CVLT-II score across AD patient subgroups**.

Variables		Low volume group (*n* = 37)	High volume group (*n* = 43)	*t*	*p*-value
Gray matter volume (mm^3^)		674.15 ± 35.48 *	728.12 ± 41.15	6.229	<0.001
Gray matter volume percentage (%)		27.63 ± 2.14 *	32.17 ± 2.09	9.581	<0.001
MMSE (points)		16.32 ± 2.01 *	20.04 ± 2.22	7.804	<0.001
CVLT-II scores					
	Immediate memory (minutes)	9.47 ± 1.32 *	13.66 ± 1.16	15.113	<0.001
	Delayed recall (points)	1.33 ± 0.28 *	1.96 ± 0.34	8.955	<0.001
	Cued recall (points)	3.43 ± 0.92 *	5.12 ± 1.01	7.774	<0.001
	Long-delayed recognition (points)	3.30 ± 1.01 *	6.43 ± 1.45	11.025	<0.001

Note: MMSE, mini-mental state examination, *p < 0.05 *vs* high volume.

### Correlation Between Gray Matter Volume/Gray Matter Volume Percentage and Cognitive and Memory Functions Across AD Patients

**Fig. 4.**
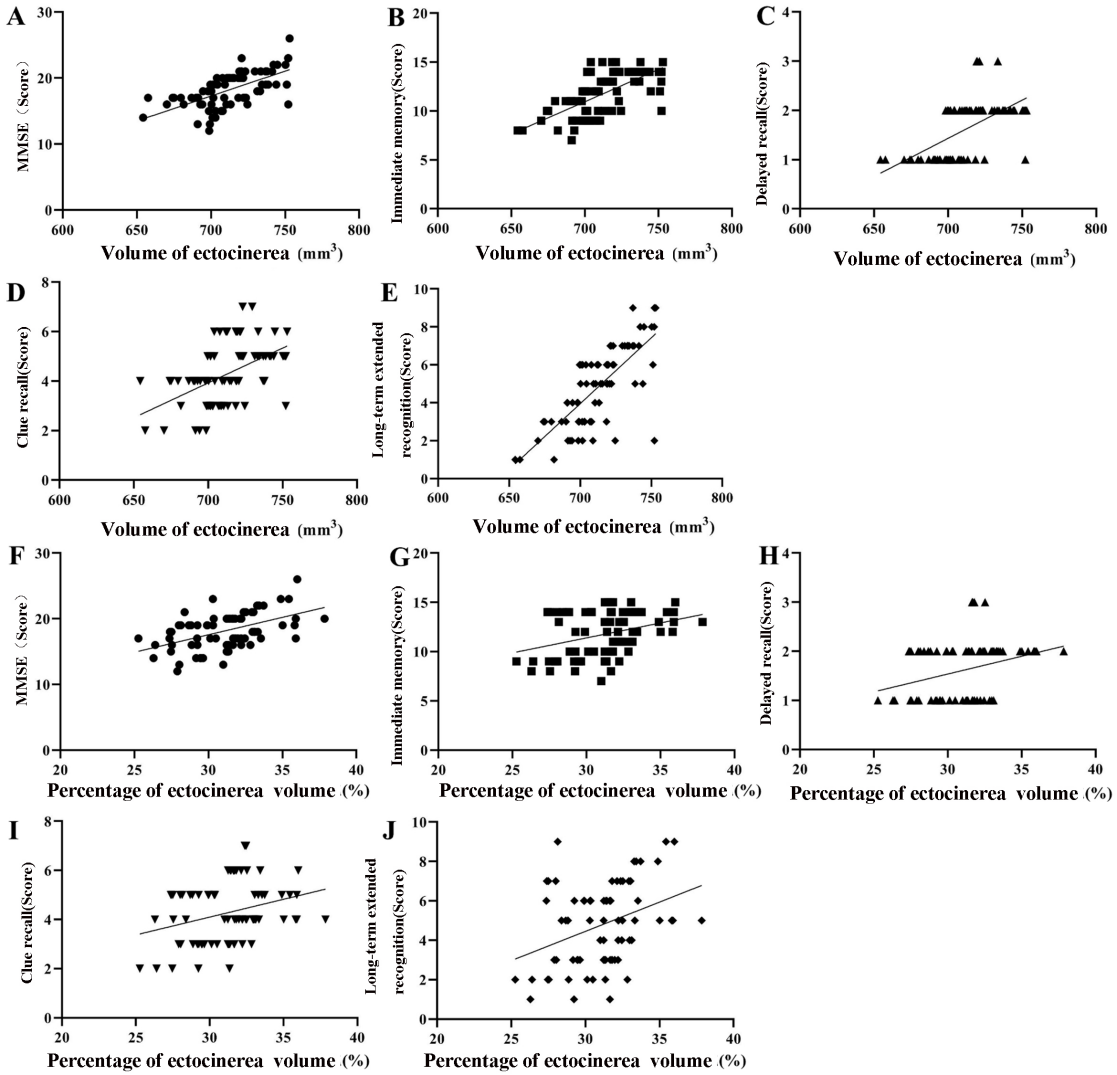
**A correlation between brain gray matter volume, brain gray matter volume percentage, MMSE score, and CVLT-II scores in AD patients**. (A,F) Correlation between brain gray matter volume, brain gray matter volume percentage and MMSE score. (B,G) Correlation between brain gray matter volume, brain gray matter volume percentage and immediate memory score. (C,H) Correlation between brain gray matter volume, brain gray matter volume percentage and delayed recall score. (D,I) Correlation between brain gray matter volume, brain gray matter volume percentage and cued recall score. (E,J) Correlation between brain gray matter volume, brain gray matter volume percentage and long-term delayed recognition score. (MMSE, Mini-Mental State Examination; CVLT-II, California Verbal Learning Test-Second Edition score).

A significant correlation was found between gray matter volume/percentage of gray matter volume and MMSE scores, as well as scores for immediate memory, delayed recall, cued recall, and long-delayed recognition (*p* < 0.001, Fig. 4, Table 5).

**Table 5.** **A correlation between brain gray matter volume/gray matter volume percentage and cognitive and memory functions across AD patients**.

Variable	Gray matter volume		Gray matter percentage	
	r	*p*-value	r	*p*-value
MMSE (points)	0.384	<0.001	0.584	<0.001
Immediate memory (minutes)	0.334	<0.001	0.319	<0.001
Delayed recall (points)	0.308	<0.001	0.299	<0.001
Cued recall (points)	0.251	<0.001	0.257	<0.001
Long-delayed recognition (points)	0.333	<0.001	0.298	<0.001

Note: MMSE, mini-mental state examination.

### Predictive Value of Gray Matter Volume/Gray Matter Volume Percentage for Severe Cognitive Impairment in AD Patients

Among AD patients, 33 cases (41.25%) had mild cognitive impairment, 26 cases (32.50%) had moderate cognitive impairment, and 21 cases (26.25%) had severe cognitive impairment. The gray matter volumes for patients with mild, moderate, and severe cognitive dysfunction were (736.54 ± 21.84) mm^3^, (695.32 ± 22.43) mm^3^, and (660.41 ± 20.63) mm^3^, respectively. However, the gray matter volume percentages were (33.41 ± 2.48)%, (29.42 ± 2.31)%, and (25.63 ± 2.14)%, respectively. The differences between the impairments were statistically significant (F value = 81.279, 72.390, *p* < 0.001).

**Fig. 5.**
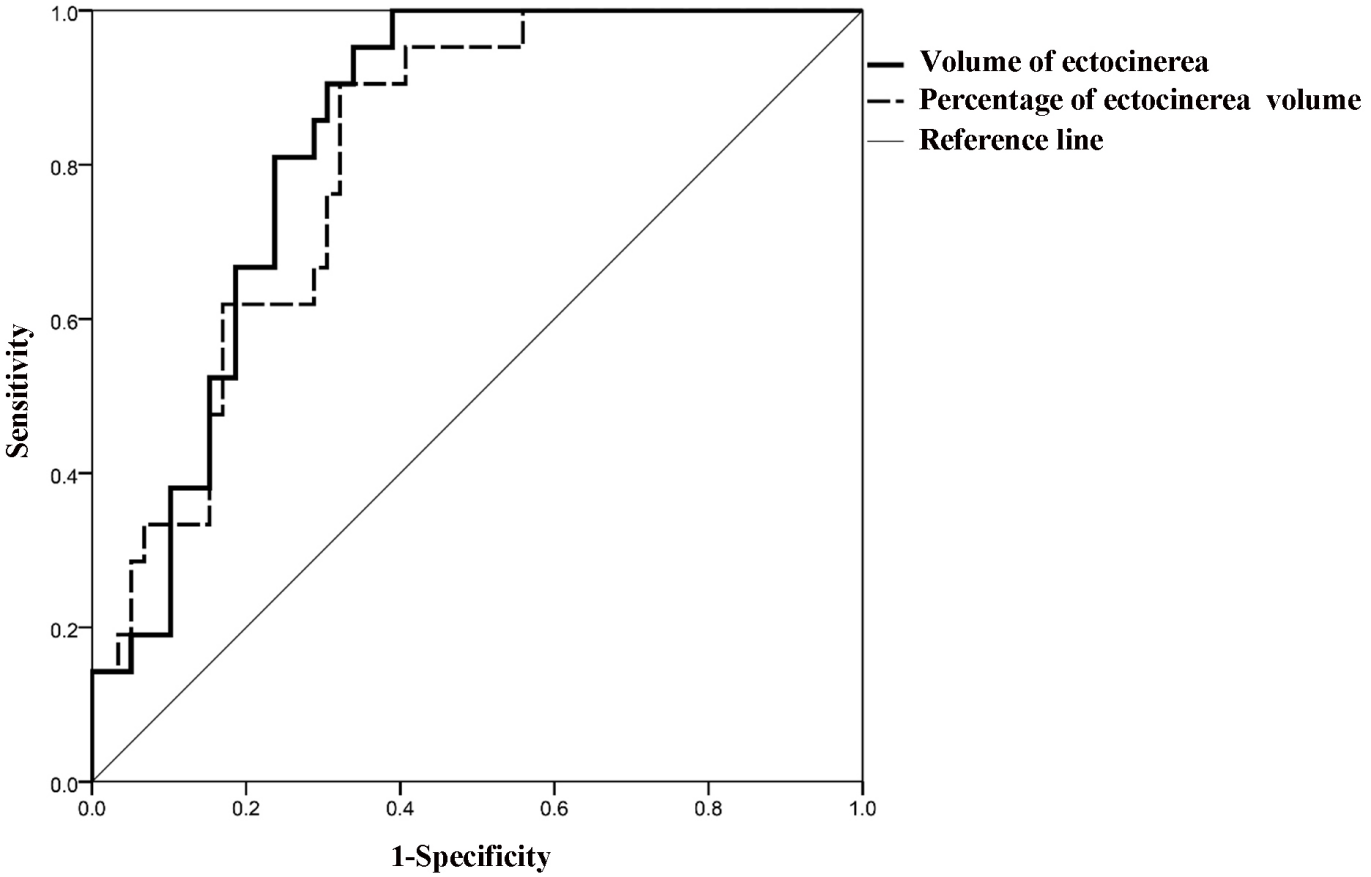
**Receiver operating characteristic (ROC) curve analysis of gray matter volume/gray matter volume percentage for predicting severe cognitive impairment in AD patients**.

ROC curve analysis was performed to evaluate the predictive value of gray matter volume and gray matter percentage for severe cognitive impairment, using cognitive impairment as the state variable and gray matter percentage as the test variable. The AUC values for gray matter volume and gray matter volume proportion were 0.833 (95% CI: 0.747~0.919) and 0.810 (95% CI: 0.715~0.904), respectively (*p* < 0.001). The optimal ROC thresholds for predicting severe cognitive dysfunction were 672.615 mm^3^ and 26.377%, for gray matter volume and gray matter volume proportion, respectively, with sensitivities of 95.24% and 90.48%, and specificities of 66.10% and 67.80% (Fig. 5).

## Discussion

MRI is commonly used for brain imaging due to its high resolution, enabling clear identification of cerebral infarction, brain atrophy, cerebral hemorrhage, and other pathological lesions compared to other imaging examinations [15]. AD is a common neurodegenerative disease in middle-aged and elderly individuals. It is characterized by the abnormal deposition of β-amyloid protein, which forms senile plaques, resulting in memory and cognitive impairment. Additionally, excessive phosphorylation of au protein leads to neurofibrillary tangles and neuronal loss, accompanied by glial cell proliferation, leading to AD pathogenesis [16].

While hippocampal lesions have been associated with memory loss and cognitive impairment in AD patients, recent studies underscore the involvement of gray matter in information processing, memory, and cognitive function [17, 18, 19]. Gray matter is one of the important components of the brain. It comprises densely packed neuronal cell bodies and is distributed across the cortical surface and deeper brain structures. Furthermore, it serves as the region for higher cognitive functions, such as thought processing, memory, and perception. In particular, the gray matter of the cerebral cortex plays a key role in these cognitive processes [20, 21].

This study selected AD patients, MCI patients, and healthy adults to analyze and compare gray matter volume and cognitive function across these groups. The results showed that MCI patients had reduced gray matter volume primarily in the temporal lobe and insular cortex, whereas AD patients exhibited reduced gray matter volume in the cortex, bilateral basal ganglia, and thalamus. Additionally, the gray matter volume, gray matter percentage, MMSE scores, and CVLT-II scores were substantially reduced in AD patients than the MCI patients, which was consistent with previous findings [22]. These observations indicate that compared with healthy people, both AD and MCI patients had decreased cognitive and memory function associated with gray matter volume, with AD patients demonstrating a more significant decline.

The decrease in gray matter volume in AD patients may be due to progressive brain atrophy, which initially affects the olfactory cortex and subsequently spreads to other cortical structures, resulting in decreased gray matter volume. Furthermore, confounding factors such as AD medication effects, lifestyle habits, and associated vascular complications may also affect gray matter decline [23]. However, the precise mechanism underlying these changes remains complex and needs further exploration at the molecular levels.

Studies on chronic mental illness have reported that these patients experience reduced gray matter volume and cognitive impairment, indicating that cognitive dysfunction is closely linked to abnormal changes in gray matter [24, 25]. In this study, AD patients were divided into low volume and high-volume groups based on their gray matter volume, and their cognitive and memory functions were analyzed. The results showed that the low-volume group had significantly reduced gray matter volume, gray matter percentage, MMSE scores, and CVLT-II scores than those in the high-volume group. These findings indicate that in the AD patient, the reduction of gray matter volume is strongly linked to impaired cognitive and memory functions. Further correlation analysis between gray matter volume, MMSE scores, and memory function scores in AD patients showed a positive correlation between gray matter volume and immediate memory, delayed recall, cued recall, and long-delayed recognition scores, which aligns with a previous study [26], indicating that gray matter volume in AD patients was closely related to their cognitive and memory functions. However, the correlation coefficient r was around 0.3, indicating a relatively weak correlation. This low correlation coefficient may be due to the small number cases collected. Since gray matter is widely distributed throughout the brain, studies have associated reduced gray matter volume with cognitive impairment and cognitive decline [27, 28].

This study revealed that the AUCs for gray matter volume and gray matter volume percentage for predicting severe cognitive impairment were 0.833 and 0.810, respectively, with sensitivities of 95.24% and 90.48%, and specificities of 66.10% and 67.80%, indicating that changes in gray matter volume serve as a valuable predictor of severe cognitive impairment in AD patients. As the main structural component of the cerebral cortex, gray matter plays a vital role in cognitive and behavioral processes through its complex neural interactions. Furthermore, located on the outer layer of the cerebral cortex, gray matter supports information storage and processing through synaptic interactions with other neurons. Therefore, decreasing gray matter volume significantly impacts cognitive and memory functions. MRI has shown strong diagnostic potential in evaluating changes of gray matter volume in AD patients with severe cognitive impairment, providing objective data for clinical assessment of cognitive impairment. Furthermore, these observations can contribute to the development of artificial intelligence-based diagnostic models for neurological diseases.

However, this study is limited by its single-center, retrospective design and small sample size, which may influence the generalizability of the findings. Future multi-center, prospective studies are required to explore the relationship between brain gray matter volume changes and AD progression.

## Conclusion

MRI is a useful tool for evaluating gray matter volume in AD patients. The changes in gray matter volume detected by MRI are strongly linked to cognitive and memory functions and serve as a reliable predictor for severe cognitive impairment in AD patients. Additionally, it provides a strong imaging basis for identifying AD patients at risk of severe cognitive impairment.

## Availability of Data and Materials

All experimental data included in this study can be obtained by contacting the Jie Zhou if needed.
